# A modular vaccine platform merging the rapid development of genetic vaccines with the immunogenicity of virus‐like particles

**DOI:** 10.1002/ctm2.70560

**Published:** 2025-12-22

**Authors:** Adam F. Sander, Cyrielle Fougeroux

**Affiliations:** ^1^ Centre for Translational Medicine and Parasitology, Department for Immunology and Microbiology Faculty of Health and Medical Sciences, University of Copenhagen Copenhagen Denmark; ^2^ AdaptVac Aps Copenhagen Denmark

Current vaccine platforms vary in their intrinsic strengths and limitations, making their suitability dependent on pathogen‐specific features and the epidemiological setting. In the early phase of an outbreak, the ability to rapidly design, manufacture and implement a vaccine is essential. However, as the epidemic progresses – or when addressing endemic pathogens – the relative priorities may shift towards durability of protection, booster compatibility, manufacturability at global scale and affordability. Currently, no vaccine platform meets all these requirements, highlighting the need for continued innovation.

During the COVID‐19 pandemic, messenger RNA (mRNA) vaccines proved their potential by enabling an exceptionally rapid vaccine rollout.^1^ However, although these vaccines demonstrated high initial immunogenicity, the vaccine‐induced neutralizing antibody titres declined rapidly, making repeated booster immunizations necessary to maintain protection.^2^ Unfortunately, evidence suggest that the short antibody durability reflects an intrinsic property of the mRNA platform rather than an antigen‐specific phenomenon.^3^

In contrast, a single dose of a human papillomavirus capsid virus‐like particle (cVLP) vaccine can provide long‐lasting protection by inducing durable antibody responses.^4^ This capacity for sustained humoral immunity is unique among subunit vaccines and is attributed to the dense, ordered, and repetitive epitope display characteristic of cVLPs, which efficiently drives robust germinal‐centre reactions and long‐lived plasma cell formation.^5^

Supporting this concept, we previously developed a modular Tag/Catcher protein‐based cVLP system that enabled the production of a non‐adjuvanted COVID‐19 vaccine which in a phase III clinical trial demonstrated non‐inferiority to the licensed mRNA vaccine Comirnaty (NCT05329220). However, cVLP vaccines have similar manufacturing timelines as other protein‐based vaccines, making frequent antigen updates to keep pace with emerging viral variants challenging.

Therefore, to combine the immunological properties of cVLPs with the manufacturing advantages of genetic vaccines, we developed a modular platform, in which co‐delivery of gene sequences encoding the target antigen and a self‐assembling cVLP enables in vivo formation of cVLP displaying the antigen at high density (Figure 1). Using the clinical‐stage malaria transmission‐blocking Pfs25 antigen as our model, we could demonstrate that this strategy enhances both humoral and cellular immunity, providing a dose sparing potential.

**FIGURE 1:**
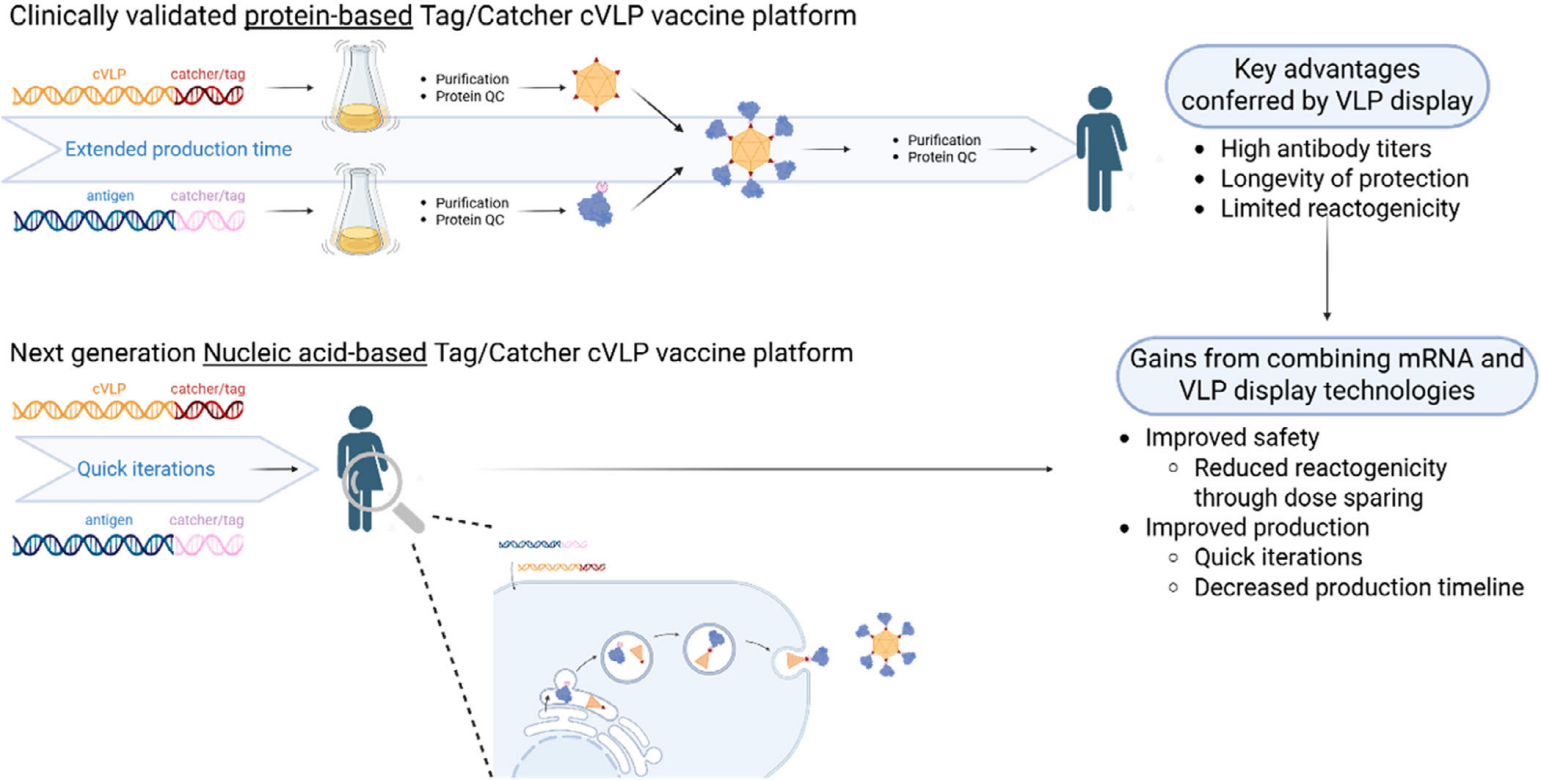
The next‐generation nucleic acid‐based Tag/Catcher capsid virus‐like particle (cVLP) vaccine platform combines the advantages of the messenger RNA (mRNA) and cVLP platform technologies, enabling rapid production, high and durable immunogenicity and improved safety through dose sparing.

## 1 KEY TECHNICAL INNOVATION

The platform is based on a genetically launched (mRNA or DNA) system encoding two components: a cVLP scaffold and a target antigen. Each component is genetically fused to a complementary split‐protein (Tag or Catcher) binding partner, enabling spontaneous formation of covalent linkages between the cVLP and the antigen.^6^ After vaccination, host cells express and assemble both components into antigen‐decorated cVLPs, which are finally secreted into the extracellular space.

By utilizing the Tag/Catcher conjugation system, this approach provides a modular platform enabling diverse combinations of cVLP scaffolds and antigens to be mixed and matched, eliminating the need for redesigning fusion constructs for each new target. The first‐generation mRNA vaccines classically express antigens in monomeric or minimally multimeric forms. However, this system enables the delivery of antigens in a highly ordered, repetitive and particulate format, which enhances the magnitude and potentially the durability of immune responses.

### 1.1 Major findings and translational relevance

1. **Enhanced humoral responses**:
  Mice immunized with the mRNA encoding for the cVLP vaccine induced higher antigen‐specific antibody titres than those receiving mRNA encoding for the soluble antigen. High antibody titres are usually associated with increased transmission‐blocking efficacy and may contribute to higher protection.
2. **Improved functional activity**:
  Anti‐Pfs25 antibodies induced by the cVLP mRNA vaccine demonstrated superior transmission‐blocking efficacy, thus confirming the increased functional capacity of the vaccine‐induced humoral response.
3. **Dose‐sparing effect**:
  Low doses of mRNA encoding for the cVLP‐displayed Pfs25 induced equivalent immune responses compared to higher doses of mRNA encoding soluble antigen, thus demonstrating a dose‐sparing potential. This feature could improve vaccine accessibility in resource‐limited settings and help mitigate dose‐dependent reactogenicity of mRNA vaccines.
4. **Prolonged antibody persistence**:
  Antibody responses generated by cVLP‐displayed Pfs25 waned more slowly than those induced by mRNA encoding the soluble antigen, consistent with the cVLP architecture's capacity to induce long‐lasting humoral immunity.
5. **Augmented cellular immunity**:
  The genetically launched cVLP system also enhanced antigen‐specific CD8⁺ T‐cell responses, first demonstrated with plasmid DNA immunization and later confirmed for mRNA‐based vaccines (unpublished). While antibodies remain the primary correlate of protection for malaria transmission‐blocking vaccines, strong cellular immunity may be crucial for other vaccines.
6. **Modular and versatile design**:
  The Tag/Catcher‐based genetic assembly system enables modular pairing of cVLP scaffolds and antigens, facilitating rapid vaccine development against diverse targets.

### 1.2 Translational potential and clinical outlook

From a clinical‐translational perspective, this study represents an important advancement in vaccine engineering by combining the rapid development and adaptability of genetic platforms with the potent and durable immune stimulation of cVLPs. The technology provides a modular ‘plug‐and‐play’ approach to allow genetically encoded antigens to be presented as cVLP, which we have shown can have a substantial impact on the elicited immune responses. From a vaccine deployment perspective, the use of this technology could translate into improved efficacy and potentially reduced antigen dose requirements or dosing frequency. These features are especially relevant for malaria transmission‐blocking vaccines, where achieving durable, high‐titre antibody responses remains a major obstacle. The combined strengths of this novel technology also hold great potential for rapid responses to emerging pathogens.

However, current data are limited to animal models, and immune responses may differ in humans. Therefore, key questions must be addressed during the clinical translation. In particular, human clinical trials will be required to assess whether the cVLP antigen display confer enhanced antibody durability. In addition, as observed with first‐generation mRNA vaccines, increased immunogenicity can be accompanied by transient reactogenicity, underscoring the need for a well‐balanced safety profile. Therefore, it is essential to evaluate the reactogenicity and safety of in vivo–assembled cVLPs.

If clinically validated, this novel technology could accelerate the development of durable, scalable and cost‐effective vaccines for both existing and emerging pathogens, and could be combined with ongoing advances in genetic vaccine delivery, sequence engineering, thermos‐stabilization and process optimization.

## CONFLICT OF INTEREST STATEMENT

Cyrielle Fougeroux and Adam F. Sander are listed as co‐inventors on a patent application covering the nucleic acid–based delivery of a modular cVLP vaccine platform (P5856PC00) and are shareholders of AdaptVac, a company developing and commercializing VLP display technologies.
